# Side effects of Pfizer/BioNTech (BNT162b2) COVID-19 vaccine reported by the Birzeit University community

**DOI:** 10.1186/s12879-022-07974-3

**Published:** 2023-01-05

**Authors:** Abdallah Damin Abukhalil, Sireen Sultan Shatat, Raya Riyad Abushehadeh, Ni’meh Al-Shami, Hani A. Naseef, Abdullah Rabba

**Affiliations:** Pharmacy Department, Faculty of Pharmacy, Nursing and Health Professions, Birzeit University, Birzeit, West Bank Palestine

**Keywords:** Pfizer/BioNTech COVID-19 vaccine, mRNA COVID-19 vaccine, Post-vaccination side effects, Palestine

## Abstract

**Background:**

The Pfizer BioNTech COVID-19 vaccine was the first to receive emergency authorization and approval from the FDA. Therefore, it is preferred by most recipients; however, many people are concerned about the vaccine’s side effects. At the time of the study, December 2021, Palestine lacked a national reporting system for monitoring adverse vaccine effects. Therefore, this study investigates the post-vaccine adverse events following the Pfizer/BioNTech COVID-19 Vaccine administration in Palestine and identifies the occurrence, extent, and severity among university staff, employees, and students at Birzeit University.

**Method:**

A questionnaire-based retrospective cross-sectional study was conducted using a university website (Ritaj), social media platforms (e.g., Facebook and Telegram), and in-person interviews. The Chi-square, Fisher’s exact, and McNemar’s tests were used to investigate significant relationships. Data were analyzed using SPSS version 22.

**Results:**

In total, 1137 participants completed the questionnaire, 33.2% were males, and the mean age was 21.163 years. All participants received at least one dose of the Pfizer-BioNTech COVID-19 vaccine. Approximately one-third of participants reported no adverse effects after receiving the first, second, or third doses (34%, 33.6%, and 32.5%, respectively). The most commonly reported adverse events were fever, chills, headache, fatigue, pain and swelling at the injection site, muscle pain, and joint pain. Allergic reactions were reported by 12.7% of the participants; furthermore, participants with a history of allergy or anaphylaxis before vaccination had a significantly higher tendency for post-vaccination allergic reactions. Eight participants reported rare side effects, including 7 (0.6%) cases of thrombocytopenia and one (0.1%) case of myocarditis. Males aged less than 20 years and smokers were significantly less likely to complain of adverse events. The number of reported side effects was significantly higher after the second vaccine dose than after the first dose. Finally, participants infected with COVID-19 before vaccination was significantly associated with side effects such as fever, chills, shortness of breath, and persistent cough.

**Conclusion:**

In this study, the most common post- BNT162b2 Vaccination reported self-limiting side effects similar to those reported by Pfizer/BioNTech Company. However, higher rates of allergic reactions were reported in this sample. Rare side effects, such as thrombocytopenia and myocarditis, were reported by 8 participants. COVID vaccines have been developed at an accelerated pace, and vaccine safety is a top priority; therefore, standard monitoring through a national adverse event reporting system is necessary for safety assurance. Continuous monitoring and long-term studies are required to ensure vaccine safety.

**Supplementary Information:**

The online version contains supplementary material available at 10.1186/s12879-022-07974-3.

## Introduction

COVID-19 infectious disease, the causative of the Coronavirus pandemic, is caused by different mutated types of Severe Acute Respiratory Syndrome Coronavirus 2(SARS-COV-2). This novel virus first appeared in December 2019 in China and later spread worldwide. Globally, as of May 21, 2022, over 524 million confirmed cases of COVID-19, including over 6.27 million deaths. In Palestine, as of May. 21, 2022, approximately 657,456 reported cases of COVID-19 and a total of 5659 deaths based on the department of health [1].

Worldwide mass efforts are in progress to develop COVID-19 vaccines and halt this pandemic by minimizing the spread and protecting human lives [2]. On 11 December 2020, the Pfizer/BioNTech vaccine became the first to get FDA emergency use authorization. On 23 August 2021, the US FDA approved the Pfizer vaccine making it the first approved COVID-19 [3, 4]. Pfizer-BioNTech Company has reported many COVID-19 vaccine reactions & adverse events, including some local reactions such as redness, swelling, and pain at the injection site and some systemic reactions such as fever, fatigue, headache, chills, vomiting, diarrhea, and new or worsening muscle/joint pain. In addition, severe adverse events have also been reported, such as appendicitis, hypersensitivity responses, acute myocardial infarction, and cerebrovascular accidents [5, 6]. Furthermore, post-marketing studies showed a slight difference in the incidence and types of side effects reported by vaccinated individuals [7, 8]. As vaccine distribution increases globally, the adverse effects should continue to be carefully monitored.

COVID-19 vaccine safety is a top priority to ensure that benefits exceed the risk. However, severe or rare adverse events may not be identified in phase 3 trials due to limited sample size, inclusion criteria, and participants’ characteristics, which may differ from the population receiving the immunization [9]. Therefore, the WHO recommended post-marketing evaluation of the safety profile of all vaccines to uncover long-term and rare adverse events associated with the vaccine [10].

The state of Palestine received the first shipment of Pfizer vaccines on March 17 2021, and as of May 21, 2022, a total of 3,720,221 vaccine doses were received, resulting in 1,768,991 (35.5/%) fully vaccinated people [11, 12]. Most countries have implemented vaccine safety monitoring and surveillance systems. For example, in the United States of America, the Vaccine Adverse Event Reporting System has been implemented. In the United Kingdom, the Yellow Card Scheme facilitates the early detection, investigation, and analysis of adverse events following vaccination (AEFIs) and adverse events of special interest (AESIs). Currently, Palestine lacks a national reporting system for monitoring vaccine adverse effects. Adverse events reporting and tracking systems in Palestine are essential as various vaccinations became available and approved with an Emergency use status [13].

This study investigates the post-vaccine adverse events following the Pfizer/BioNTech COVID-19 Vaccine administration in Palestine to alleviate the incomplete clinical trial gap and support the national strategic readiness and response plan. Furthermore, to identify the occurrence, extent, and severity of adverse events among university staff, employees, and students at Birzeit University and compare the incidence of these side effects between the first, second, and third doses and the data published by Pfizer company. Finally, this study aimed to predict the post-vaccination side effects based on individual predisposing factors such as age, gender, smoking status, food/drug allergy, comorbidities, and COVID-19 infection before vaccination.

## Materials and methods

### Study design and sample

A questionnaire-based retrospective cross-sectional study was conducted at Birzeit University in Palestine, which started the vaccination of the university community in September 2021, from December 13, 2021, to March 29, 2022. The study included participants aged 18 years and older who received at least one dose of the Pfizer/BioNTech COVID-19 Vaccine. The questionnaire was distributed through the university website (Ritaj), social media platforms (e.g., Facebook, Telegram, etc.), and in-person interviews. 1496 participants were included in this study, while 375 were excluded because they refused to participate, had an incomplete response to the questionnaire, or received other types of COVID-19 Vaccines.

### Questionnaire (survey)

The questionnaire was prepared in English, following a thorough literature review [14, 15]. The researchers examined and evaluated the questionnaire elements at different sessions. Furthermore, it was expertly translated into Arabic since it is the official language of the target population (Additional file 1).

A pilot study was conducted to confirm questionnaire consistency among 32 vaccinated COVID-19 individuals, who were asked to complete the questionnaire and provide feedback on its clarity, relevance, and construction. These pilot study responses were not included in the formal evaluation, and modifications were made to the final Arabic draft based on the participants’ reviews.

The questionnaire included five sections with 27 questions formulated as open- and closed-ended multiple-choice questions besides two short essay questions. The first section included nine questions concerning demographic information, such as gender, age, weight, height, employment, chronic diseases, allergic reactions, and smoking status. The second section included four questions about infection status with SARS-Cov-2 before vaccination. The third section consisted of 4 questions on the COVID-19 vaccines, such as the type, the number of doses received post-vaccination counseling and allergic reactions after vaccination. The fourth section, “Pfizer-BioNTech side effects,” consists of a list of 23 possible side effects divided into two categories as local or systemic adverse events according to the world health organization’s global manual on surveillance of adverse events following immunization [16]. Adverse events were chosen using the WHO, CDC leaflet, and reports published by randomized control trials on the Pfizer-BioNTech vaccine [5, 17]. The local adverse events compromised of pain at the injection site, and swollen armpit glands, while systemic adverse events include fever, chills, headache, increase in blood pressure, increase in heart rate, shortness of breath, persistent cough, chest pain, voice hoarseness, dizziness, nausea, vomiting, diarrhea, abdominal pain, tiredness and fatigue, muscle pain (myalgia), joint pain, swollen ankles and feet, and sleep disturbances. In addition to side effects that were self-noticed as menstrual cycle changes and tinnitus. Also, space was provided for reporting other uncatalogued side effects which may be experienced. Other questions in this section included the onset and duration of experienced side effects, participant’s attitude after vaccination, and any doctor’s visit or hospital admission due to severe side effects. Finally, the last section included four questions about the infection status with SARS-Cov-2 after vaccination.

### Statistical analysis

The data were analyzed using IBM Statistical Package for the Social Sciences (SPSS) version 22. Descriptive statistics were used to analyze the data. Frequencies and percentages were measured for categorical data, whereas means and standard deviations were measured for continuous data to be used as descriptive statistics. First, questions were recoded and categorized; height and weight were computed to BMI and then categorized based on BMI classification: underweight (below 18.5), normal weight (18.5–24.9), overweight (25.0–29.9), and obese (≥ 30.0) [18]. Next, ages were divided into two groups (lower than 20, 20, and more years old).

Chi-square (χ^2^) or Fisher’s exact tests were performed to investigate the association between participants’ demographics and post-vaccination side effects for the first and second doses. Chi-square (χ^2^) or Fisher’s exact tests were also applied to investigate the association between participants’ demographics and onset plus side effects duration. In addition, McNemar’s tests were conducted to compare the incidence of each side effect between the first and second shots. All inferential tests were performed considering a confidence interval (CI) of 95% and a significance value of p < 0.05.

### Ethical considerations

All participants were given a brief introduction to the study’s objectives and were asked to provide their informed consent before completing the questionnaire. This consent procedure was approved by the Ethics Committee of the Research Ethics Committee at the Faculty of Pharmacy, Nursing, and Health Professions, Birzeit University (reference number BZU-PNH-2103).

## Results

### Demographic characteristics

This study included 1137 participants from Birzeit University in Palestine. All participants received at least one dose of the Pfizer-BioNTech COVID-19 Vaccine. The mean age of the participants was 21.163 years ± 5.361, with 63.2% older than 20 years. In addition, 66.8% were females, 91.8% were healthy without chronic diseases, 26.7% were smokers, and 14.4% had drug/food allergies (Table1).

**Table 1:** Demographic characteristics of the participants (N = 1137)

Variable	Category	n (%)
Gender	Male	378 (33.2%)
	Female	759 (66.8%)
Age	(mean ± SD)	21.163 ± 5.361
	Less than 20	418 (36.8%)
	20 and more	719 (63.2%)
Participants	University employee	55 (4.8%)
	Student	1082 (95.2%)
BMI	Underweight (< 18.5)	92 (8.1%)
	Normal (18.5–24.9)	735 (65.1%)
	Overweight (25.0–29.9)	236 (20.9%)
	Obese (≥ 30)	66 (5.8%)
Smoking	Smoker	304 (26.7%)
Comorbidities	Yes	93(8.2%)
Allergies	Drug / food	164 (14.4%)
	Post-Pfizer Vaccine	144 (13%)
Number of vaccine doses	One dose	110 (9.7%)
	Two doses	876 (77.0%)
	Three doses	151 (13.30%)
Vaccine Side effects Counseling	Received Counsel by Health care provider	583 (51%)

### COVID-19 infection

**Fig. 1:**
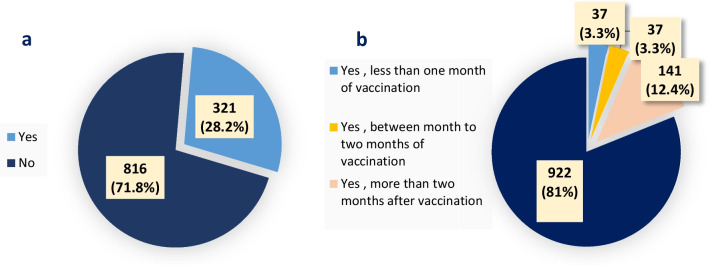
COVID-19 infection Status (N = 1137). Pre-vaccination (**A**). Post-vaccination (**B**)

Figure 1 shows the participants’ COVID-19 infection status before and after Pfizer-BioNTech COVID-19 Vaccination. A total of 321 (28.2%) participants were infected with SARS-Cov2 before COVID-19 vaccination. As a result, 15 (4.7%) were admitted to the hospital, and 10 (66.7%) were hospitalized for less than 7 days. On the other hand, 215 (19%) were infected post-vaccination, and 11 (5.1%) were hospitalized, with 8 (72.7%) hospitalized for less than 7 days.

### Post-vaccination side effects

**Fig. 2:**
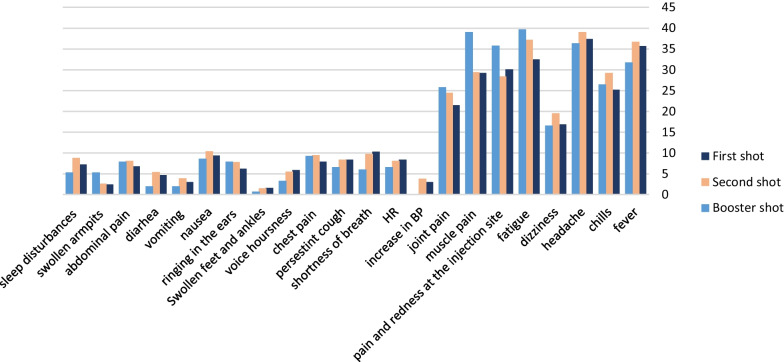
Side effects of Pfizer (BioNTech) vaccine reported by our study

Approximately one-third of participants reported no adverse effects after receiving the first, second, or third dose of the Pfizer vaccine, with 34%, 33.6%, and 32.5%, respectively. Participants who received more than one dose experienced more side effects. The most commonly reported adverse effects were fever, chills, headache, fatigue, pain and swelling at the injection site, muscle pain, and joint pain. Allergic reactions following vaccination, such as allergic skin reactions (itching, burning, and rash), angioedema, shortness of breath, coughing, and significant swelling of the tongue or lips, were reported by 144 (12.7%) participants (Fig. 2). Rare and severe side effects that require medical attention were reported by eight participants, including one (0.1%) case of myocarditis and 7 (0.6%) cases of thrombocytopenia.

**Fig. 3:**
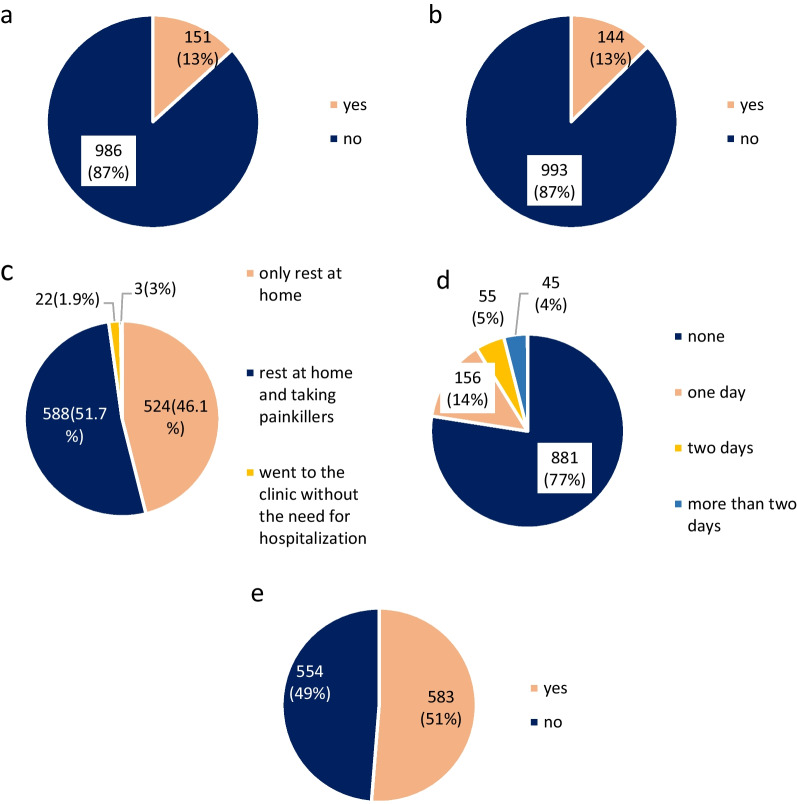
Post-vaccination information. Shoulder injury related to vaccine administration (**A**). Post-vaccination allergic reaction (**B**). Action to relieve symptoms that appeared after receiving the vaccine (**C**). Number of days missed of work/university due to vaccine side effects (**D**). Counseling about post-vaccination side effects by a healthcare provider (**E**)

A relatively high percentage (13%) of participants reported shoulder injury related to vaccine administration (SIRVA) (Fig. 3A). Furthermore, a considerable percentage (13%) of the participants experienced at least one type of allergic reaction after receiving the Pfizer BioNTech vaccine (skin rash, persistent cough, shortness of breath, angioedema, swelling of the tongue or lips) (Fig. 3b). In addition, 51.7% of participants took pain relievers post-vaccine for side effects management (Fig. 3c). Furthermore, 45 (4%) missed more than two days of work or school due to post-vaccination side effects or allergic reactions to the vaccine (Fig. 3d), and 49% of vaccine recipients were not counseled about the vaccine’s side effects (Fig. 3e).

As shown in Table 2, McNemar’s test results revealed that the proportion of respondents who reported systemic adverse events after the second vaccine dose and did not report after the first dose was statistically significantly increased (p-value < 0.05). These adverse events include; chills (14.2%), Chest pain (6%), Dizziness (9.9%), Ringing in the ears (Tinnitus) (4.9%), tiredness and fatigue (12.5%), Joint pain (10%), over sleepiness, and decreased sleep quality (4%), and (9.5%) of females reported menstrual cycle changes after the second dose only.

**Table 2:** Reported adverse events Post-Pfizer vaccine First and Second dose (N = 1027)

Adverse event	Reported after 1st dose	Reported after 2nd dose		P- value
		Yes	No	
No symptoms	Yes	216 (21.0)	133 (13.0)	0.853
	No	129 (12.6)	549 (53.5)	
Local adverse events				
Pain or swelling at the injection site	Yes	232 (22.6)	84 (8.2)	0.068
	No	61 (5.9)	650 (63.3)	
Swollen armpit glands	Yes	11 (1.1)	11 (1.1)	0.441
	No	41.6 (1.6)	989 (96.3)	
Systemic adverse events				
Fever	Yes	214 (20.8)	150 (14.6)	0.498
	No	163 (15.9)	500 (48.7)	
Chills	Yes	154 (15.0)	101 (9.8)	0.005
	No	146 (14.2)	626 (61.0)	
Headache	Yes	245 (23.9)	136 (13.2)	0.221
	No	158 (15.4)	488 (47.5)	
Increase in blood pressure	Yes	11 (1.1)	18 (1.8)	0.185
	No	28 (2.7)	970 (94.4)	
Increase in heart rate	Yes	29 (2.8)	50 (4.9)	0.769
	No	54 (5.3)	894 (87.0)	
Shortness of breath	Yes	37 (3.6)	55 (5.4)	0.463
	No	64 (6.2)	871 (84.8)	
Persistent Cough	Yes	34 (3.3)	44 (4.3)	0.475
	No	52 (5.1)	897 (87.3)	
Chest pain	Yes	36 (3.5)	37 (3.6)	0.016
	No	62 (6.0)	892 (86.9)	
Voice hoarseness	Yes	19 (1.9)	38 (3.7)	1.000
	No	37 (3.6)	933 (90.8)	
Dizziness	Yes	98 (9.5)	71 (6.9)	0.023
	No	102 (9.9)	756 (73.6)	
Ringing in the ears (Tinnitus)	Yes	30 (2.9)	28 (2.7)	0.017
	No	50 (4.9)	919 (89.5)	
Nausea	Yes	53 (5.2)	36 (3.5)	0.073
	No	54 (5.3)	884 (86.1)	
Vomiting	Yes	9 (0.9)	17 (1.7)	0.061
	No	31 (3.0)	970 (94.4)	
Diarrhea	Yes	25 (2.4)	21 (2.0)	0.263
	No	30 (2.9)	951 (92.6)	
Abdominal pain	Yes	41 (4.0)	27 (2.6)	0.091
	No	42 (4.1)	917 (89.3)	
Tiredness and fatigue	Yes	254 (24.7)	82 (8.0)	0.002
	No	128 (12.5)	563 (54.8)	
Muscle pain (myalgia)	Yes	191 (18.6)	105 (10.2)	0.734
	No	111 (10.8)	620 (60.4)	
Joint pain	Yes	148 (14.4)	70 (6.8)	0.012
	No	104 (10.1)	705 (68.6)	
Swollen ankles and feet	Yes	4 (0.4)	11 (1.1)	1.000
	No	11 (1.1)	1001 (97.5)	
Sleep disturbances	Yes	49 (4.8)	20 (1.9)	0.010
	No	41 (4.0)	917 (89.3)	
Menstrual cycle changes	Yes	60 (8.6)	20 (2.9)	< 0.001
	No	66 (9.5)	548 (79.0)	

*McNemar’s test

**Fig. 4:**
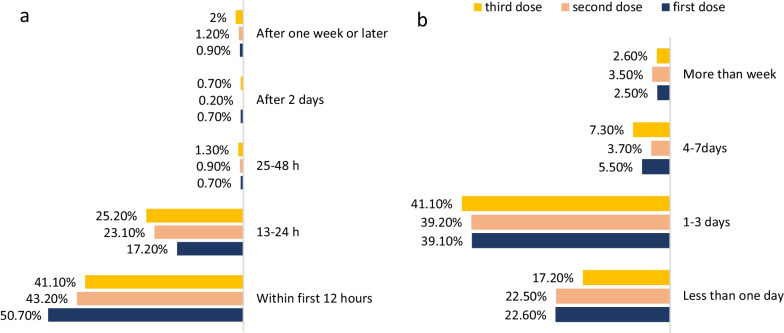
Onset and duration of side effects after Pfizer vaccination for the three doses. Onset of side effects (**A**). Duration of side effects (**B**). *First dose N = 1137 **Second dose N = 1027 ***Third dose N = 151

The onset and duration of side effects vary among vaccine doses. The onset of side effects was reported within 12 h of vaccination by 50.70%, 43.20%, and 41.10% of participants for the first, second, and third doses. Furthermore, side effects persisted from 1 to 3 days in 41.10%, 39.20%, and 39.10% for the first, second, and third vaccine doses (Fig. 4). The onset and duration of symptoms were not affected by age or gender in the three doses.

### Association of demographic characteristics of the participants and side effects after the first dose

Table 3 shows the reported adverse effects of the Pfizer vaccine and their association with participants’ demographic factors. Females were significantly more likely to complain of side effects such as fever (39.1%), chills (29.8%), headache (41.9%), fatigue (36.8%), myalgia (32.7%), pain and swelling at the injection site (34.9%) with (p-value ≤ 0.001), shortness of breath (11.6%, p-value = 0.04), dizziness (18.8%, p-value = 0.013), nausea (11.1%, p-value = 0.007), arthralgia (24.1%, p-value = 0.003) and sleep disturbances (8.8%, p-value = 0.003) after the first dose. Participants under 20 years (39%) experienced significantly fewer side effects after the first dose than those older (31.2%, p- value = 0.007). Smokers also reported fewer adverse events (40.5%, p < 0.006) compared with non-smokers (31.7%). Furthermore, participants with food and drug allergies reported significantly more adverse effects following the first vaccine dose, such as fever (47.6%, p-value = 0.001), chills (16.7%, p-value = 0.001), headache (48.2%, p-value = 0.002), increased heart rate(18.3%, p-value < 0.001), shortness of breath (20.7%, p-value < 0.001), a persistent cough (13.4%, p-value = 0.013), chest pain (12.2%, p-value = 0.028), voice hoarseness (10.4%, p-value = 0.009), dizziness (26.2%, p-value = 0.001), nausea (15.2%, %, p-value = 0.006), vomiting (6.7%, p-value = 0.006), diarrhea (8.5%, p-value = 0.011), myalgia (39.6%, p-value = 0.001), and sleep disturbances (13.4%, p-value = 0.001).

**Table 3:** The Association between Post-vaccination Adverse Events Reported by the Participants for the first shot and their predisposing factors (N = 1137)

Post-vaccination adverse events	Predisposing factors (p-value*)							
	Gender	Age	Chronic diseases	BMI	Allergy	Anaphylaxis shock	Smoking	Pre-vaccination COVID-19 infection
No symptoms	< 0.001	0.007	0.288	0.289	0.08	0.105	0.006	0.016
Local adverse events								
Pain and swelling at the injection site	< 0.001	0.058	0.526	0.283	0.010	0.011	0.049	0.057
Swollen armpit glands	0.414	0.113	1.000	0.655	0.161	1.000	0.157	0.870
Systemic adverse events								
Fever	0.001	0.543	0.79	0.140	0.001	0.079	0.437	0.001
Chills	< 0.001	0.384	0.251	0.046	0.001	0.750	0.490	0.002
Headache	< 0.001	0.390	0.039	0.525	0.002	0.027	0.141	0.014
Increase in Blood pressure	0.911	0.857	0.017	0.559	0.135	0.047	0.453	0.089
Increase in heart rate	0.051	0.078	0.041	0.216	< 0.001	< 0.001	0.923	0.141
Shortness of breath	0.04	0.157	0.005	0.098	< 0.001	< 0.001	0.777	< 0.001
Persistent cough	0.266	0.412	0.106	0.463	0.013	0.055	0.748	0.005
Chest pain	0.361	0.166	0.145	0.934	0.028	0.032	0.447	0.00
Voice hoarseness	0.381	0.254	0.011	0.114	0.009	0.015	0.553	0.024
Dizziness	0.013	0.223	< 0.001	0.941	0.001	0.001	0.905	0.170
Ringing in the ears (tinnitus)	0.057	0.746	0.140	0.683	0.085	0.031	0.720	0.245
Nausea	0.007	0.889	0.052	0.212	0.006	0.123	0.583	0.079
Vomiting	0.112	0.857	0.191	0.945	0.006	0.047	0.453	0.353
Diarrhea	0.853	0.191	0.001	0.017	0.011	0.241	0.125	0.059
Abdominal pain	0.161	0.194	0.004	0.996	0.100	0.004	0.672	0.003
Tiredness and fatigue	< 0.001	0.173	0.125	0.055	0.127	0.001	0.189	0.379
Myalgia (muscle pain)	< 0.001	0.174	0.019	0.287	0.001	0.025	0.196	0.039
Joint pain	0.003	0.290	0.436	0.280	0.047	0.054	0.934	0.001
Swollen ankles and feet	0.317	0.850	1.000	0.981	0.497	0.620	0.428	0.568
Sleep disturbances	0.003	0.221	0.533	0.470	0.001	0.328	0.309	0.969
Menstrual cycles changes		0.163		0.952	0.011	0.542	0.345	0.001

Chi-square test*

There was no statistically significant difference across BMI categories except a significant association with the incidence of chills reported more frequently in underweight participants (34.8%, p-value = 0.046). Moreover, the presence of comorbidities was statistically significant with headache (47.3%, p-value = 0.039), increase in blood pressure (p-value = 0.017), increase in heart rate (7.5%, p-value = 0.041), shortness of breath (19.4%, p-value = 0.005), voice hoarseness (11.8%, p-value = 0.011), dizziness (30.1%, p-value < 0.001), diarrhea (12.9%, p-value = 0.001), abdominal pain (14%, p-value = 0.004), and myalgia (39.8%, p-value = 0.019).

Participants infected with COVID-19 before vaccination were significantly associated with side effects such as fever (43.3%, p-value = 0.001), chills (31.5%, p-value = 0.002), headache (43%, p-value = 0.014), shortness of breath (16.5%, p-value < 0.001), a persistent cough (12.1%, p-value = 0.005), chest pain (12.8%, p-value = 0.001), abdominal pain (10.3%, p-value = 0.003), joint pain (27.7%, p-value = 0.001), menstrual cycle changes (17.8%, p-value = 0.001), voice hoarseness (8.4%, p-value = 0.024), and myalgia (33.6%, p-value = 0.039).

### Association of demographic characteristics of the participants and side effects after the second dose

**Fig. 5:**
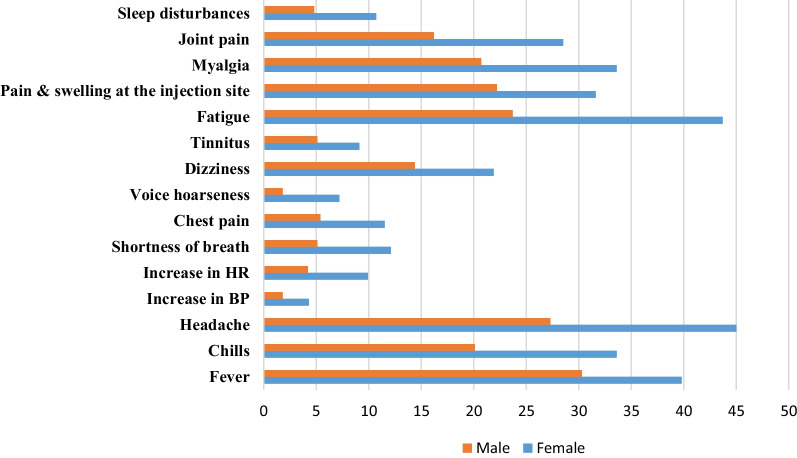
Side effects difference between males and females

Table 4 shows a significant association between side effects after the second dose and all demographic characteristics except BMI categories (p-value > 0.05). Females were more likely to complain of the most common adverse events except an increase in blood pressure and heart rate, persistent cough, chest pain, voice hoarseness, ringing in the ears, vomiting, diarrhea, abdominal pain, and swollen armpit glands, ankles, and feet (p-value > 0.05). Figure 5 shows the differences between males and females in percentages. Furthermore, there was no significant association between age group and post-vaccination except for persistent cough and menstrual cycle changes; participants younger than 20 years have a higher incidence of persistent cough (11.2%) compared to older (6.7%, p-value = 0.012). Females under 20 years experienced lower menstrual cycle changes (13.5%) compared to older (20.9%, p-value = 0.014). Add that smokers have a lower incidence of vaccine-adverse events (31.7%) than non-smokers (38.9%, p-value = 0.032). However, they experience a higher incidence of diarrhea (8.1%, p-value = 0.018) and swollen ankles and feet (3%, p-value = 0.032) compared to non-smokers (4.4% and 0.9%), respectively.

**Table 4:** The Association between Post-vaccination Side effects Reported by the Participants for the second shot and their predisposing factors (N = 1027)

Post-vaccination adverse events	Predisposing factors (p-value*)							
	Gender	Age	Chronic diseases	BMI	Allergy	Anaphylaxis shock	Smoking	Pre-vaccination COVID-19 infection
No symptoms	< 0.001	0.382	0.438	0.328	0.279	0.740	0.032	0.149
Local adverse events								
Pain and swelling at the injection site	0.002	0.852	0.993	0.545	0.007	0.937	0.641	0.144
Swollen armpit glands	0.465	0.458	0.270	0.118	0.256	0.185	0.965	0.771
Systemic adverse events								
Fever	0.003	0.618	0.145	0.379	0.010	0.086	0.472	0.092
Chills	< 0.001	0.301	0.018	0.083	0.013	0.686	0.860	0.076
Headache	< 0.001	0.527	0.035	0.868	0.003	0.196	0.248	0.717
Increase in Blood pressure	0.020	0.683	0.363	0.653	0.035	0.04	0.782	0.106
Increase in heart rate	0.002	0.443	0.003	0.811	0.039	0.02	0.408	0.512
Shortness of breath	< 0.001	0.358	< 0.001	0.144	< 0.001	< 0.001	0.712	0.024
Persistent cough	0.098	0.012	0.691	0.367	< 0.001	0.048	0.261	0.029
Chest pain	0.002	0.553	0.247	0.344	0.005	0.099	0.854	0.025
Voice hoarseness	< 0.001	0.059	1.000	0.107	0.044	0.121	1.000	0.581
Dizziness	0.005	0.398	0.028	0.443	< 0.001	0.002	0.540	0.555
Ringing in the ears (tinnitus)	0.026	0.488	0.536	0.528	< 0.001	< 0.001	0.065	0.264
Nausea	0.058	0.826	0.226	0.895	< 0.001	0.007	0.976	0.657
Vomiting	0.497	0.885	0.562	0.602	< 0.001	0.001	0.100	0.681
Diarrhea	0.401	0.559	0.038	0.017	< 0.001	0.001	0.018	0.028
Abdominal pain	0.053	0.673	0.613	0.390	< 0.001	0.004	0.831	0.007
Tiredness and fatigue	< 0.001	0.175	0.954	0.393	0.093	0.284	0.276	0.402
Myalgia (muscle pain)	< 0.001	0.672	0.115	0.122	0.025	0.205	0.709	0.164
Joint pain	< 0.001	0.276	0.154	0.277	0.079	0.026	0.837	0.564
Swollen ankles and feet	0.631	0.771	0.351	0.089	0.002	0.007	0.032	0.138
Sleep disturbances	0.002	0.610	0.008	0.117	0.001	0.026	0.505	0.891
Menstrual cycles changes		0.014		0.977	0.001	0.094	0.427	0.137

Chi-square test*

**Fig. 6:**
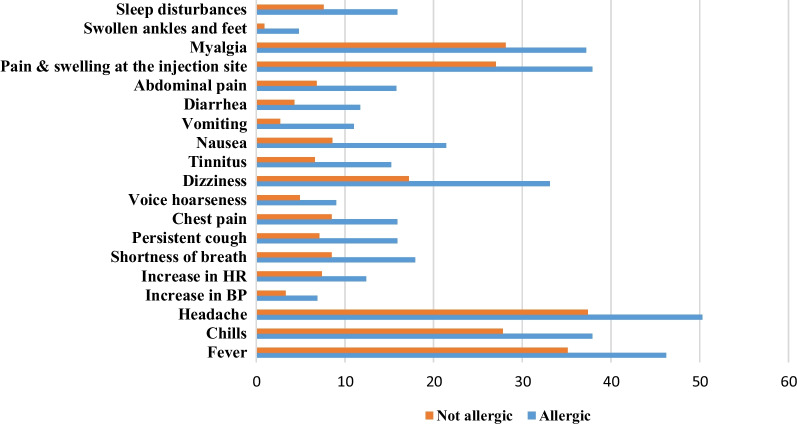
Side effects frequency among patients who suffered from drug/food allergy

There were significant associations between participants who suffered from drug/food allergy with the frequencies of all adverse events following the second vaccine shot except tiredness and fatigue, joint pain, and swollen armpit glands. The reported differences are shown in Fig. 6. Moreover, the presence of comorbidities was statistically significant with the incidence of side effects such as an increase in heart rate (16.7%, p-value = 0.003), shortness of breath (21.4%, p-value < 0.001), sleep disturbances (16.7%, p-value = 0.008), chills (40.5%, p-value = 0.018), dizziness (28.6%, p-value = 0.028), and diarrhea (10.7%, p-value = 0.038). In addition, experiencing COVID-19 infection before vaccination was significantly associated with side effects such as shortness of breath (13.3%, p-value = 0.024), a persistent cough (11.5%, p-value = 0.029), chest pain (12.9%, p-value = 0.025), diarrhea (7.9%, p-value = 0.028), and abdominal pain (11.8%, p-value = 0.007).

## Discussion

As information about the COVID-19 vaccine continues to evolve, and with the FDA approval for human use, post-marketing studies are necessary to ensure safety, efficacy, and use. Therefore, this study was conducted as a post-marketing survey of the Pfizer-BioNTech vaccine in Palestinian society. First, we investigated the incidence of side effects and reinfection rates following the administration of the Pfizer-BioNTech vaccine, then compared the incidence of these side effects between the first, second, and third doses. In addition, we predict the post-vaccination side effects based on predisposing factors.

Adverse effects were reported in more than two-thirds of the study participants. Most of the side effects were experienced within 12 h of vaccination and persisted for 1–3 days. This finding is similar to a systemic review of Pfizer-BioNTech COVID-19 Vaccine side effects and other COVID-19 vaccines, where participants suffered from post-vaccination adverse effects after the three doses, and to an Egyptian study where adverse events resolved a couple of days after onset [14, 19–21]. These side effects are related to the normal immune system’s response to the vaccine constituents resulting in the transient production of cytokines that cause inflammation in the muscles, blood vessels, and other tissues.

A wide range of common side effects, including fever, chills, headache, fatigue, pain and swelling at the injection site, muscle pain, and joint pain, were reported by participants. In addition, some side effects were reported less commonly, such as increased blood pressure, increased heart rate, vomiting, diarrhea, swollen armpit glands, swollen ankles and feet, and others. These findings are consistent with those reported in the Pfizer-BioNTech factsheet by the Food and Drug Administration (FDA) and many other studies [22, 23]. Myocarditis was reported in many published case reports, case series, and retrospective studies following COVID-19 mRNA immunization in young people (18–25 years) after receiving the second vaccination dose; however, the incidence was rare [24, 25]. Many factors must be considered when assessing the risk of myocarditis, including patient factors, medical history, and SarsCov2 infection history [26]. The Centers for Disease Control and Prevention (CDC) reported that infection with the Sars-cov2 virus increases the risk for myocarditis; therefore vaccine’s benefits in preventing COVID-19 infection, hospitalization, and intensive admissions and death exceed the potential risk of myocarditis [27]. The localized lymphadenopathy (enlarged axillary nodes) reported in this study aligns with the first Therapeutic Goods Administration’s COVID vaccine safety report of 2022, which is more common in the mRNA booster dose [28, 29]. Enlarged axillary nodes are a normal response caused by a robust vaccine-elicited immune response due to the proliferation of rapidly activated immune cells (residual effector cells) from previous doses [30, 31].

The severity of most experienced side effects reported after each of the three vaccine doses was mild to moderate and self-limiting, similar to the published results of the phase III Pfizer clinical trial, with the majority occurring after the second dose [22, 23, 32]. Interestingly, some side effects after the third dose were reported more frequently, including fatigue, pain at the injection site, myalgia, and arthralgia, which is inconsistent with the Food and Drug Administration (FDA) report on the booster dose [3].

**Fig. 7:**
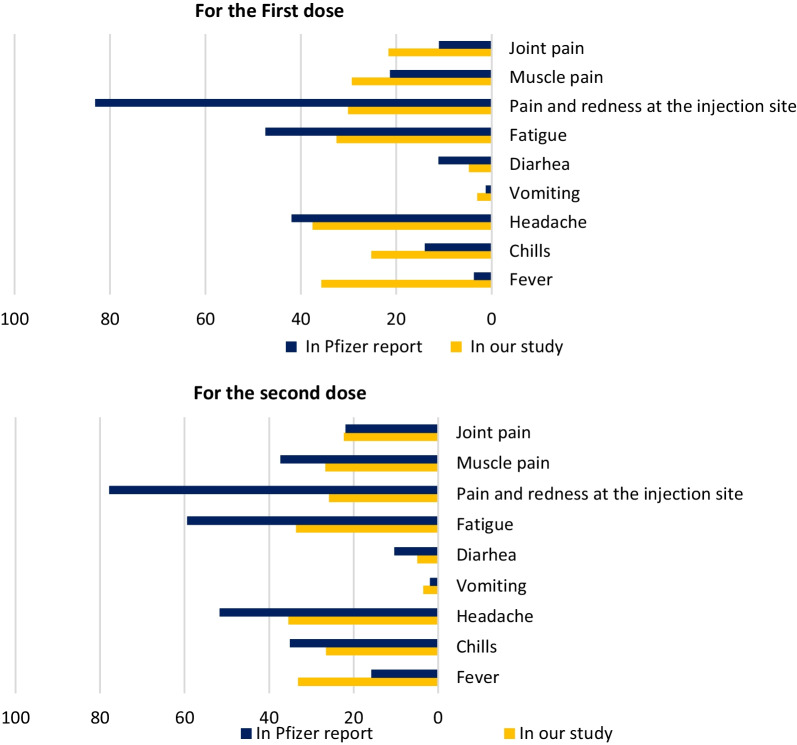
Comparison between First and second doses’ post-vaccination side effects in our study and those reported by CDC and Pfizer Company

Specific Side effects types and severity reported by participants differed slightly from the CDC, the FDA, or other studies. For example, as shown in Fig. 7, there is an increase in the occurrence of fever (35.7%, and 33.1% for first and second doses, respectively) compared to the CDC (3.7% and 15.8%). Furthermore, study participants reported less injection site pain and redness (30.1%, 25.8%) than what is reported on the Pfizer-BioNTech factsheet (83.1%, 77.8%) and other regional studies [6, 14, 33]; a similar finding was reported in a Bahraini study where the incidence of fever and pain at the injection site after the first dose (25%, 43%).

A considerable percentage (13%) of the participants experienced at least one type of allergic reaction after receiving the Pfizer BioNTech vaccine (skin rash, persistent cough, shortness of breath, angioedema, swelling of the tongue or lips) (Fig. 3d). However, anaphylactic reactions associated with vaccines are rare, and it has been reported at a rate of 1:125,000 doses for the Pfizer/BioNTech COVID-19 vaccine, perhaps due to PEGylated nanoparticles (PEG2000) as excipients [32].

Participants with a history of allergy or anaphylaxis prior to vaccination had a significantly higher tendency for post-vaccination allergic reactions and side effects caused by a stronger immune response. The injection of foreign materials triggers a non-specific inflammation caused by the activation of macrophages, dendritic cells, eosinophils, basophils, and especially innate type 2 lymphoid cells and the decreased activation threshold of effector cells (mast cells among them) to non-IgE activating factors [34]. In a cohort study to investigate the association of high-risk allergy history with allergy symptoms post COVID-19 vaccination, subjects with food and drug allergies were more susceptible to experiencing a post-vaccination allergic reaction [35].

Therefore, healthcare providers and institutions are encouraged to follow the CDC’s COVID-19 vaccination guidance, including pre-vaccination screening forms, enforcing the recommended 15-min post-vaccination observation periods, and having the necessary reserves available to handle severe allergic reactions.

The participants’ demographic data revealed a significant percentage of females, young, non-smokers, and individuals of normal weight. These findings refer to the large percentage of student participation and a higher percentage of female vs. male students at Birzeit University (62% vs. 38%, respectively) [36].

Females experienced a higher incidence of side effects after receiving the Pfizer-BioNTech vaccine. These gender variations were also found in previous vaccines, as reported by a 2019 study on allergic reactions following the 2009 flu vaccine [37]. Gender biological variations, including hormones, genes, immunologic distinctions, pharmacokinetics, and pharmacodynamics, affect vaccine response [19, 38]. The difference in immune response between males and females was demonstrated in a study by Yale University in 2020, in which the blood samples of females taken following COVID-19 infection showed a higher t-cell response than male blood samples [39]. Therefore, vaccine manufacturers should consider the higher female immune response and offer a reduced vaccine dose for females, reducing the side effects and maintaining the same efficacy or considering a later booster dose [40].

Many studies have addressed smoking, vaccinations, and the risk of COVID-19 infections. The CDC developed its recommendation for the necessity of smoker vaccination based on smokers' high risk of COVID infections. Smokers who participated in the study experienced a lower prevalence of post-COVID-19 vaccination side effects than non-smokers. This finding is supported by other studies where nonsmokers who received the COVID-19 vaccine experienced a higher incidence of pain and swelling at the injection site after the first dose than smokers [15]. Smoking could affect immunological responses, but further investigations are needed to define the effects of smoking on vaccinations and immunologic responses. A systematic review of the effect of smoking on humoral immunity to vaccine P. Ferrara demonstrated that antibody titers dropped rapidly in COVID-19 vaccinated smokers [41].

Elderly patients and patients with comorbid diseases prioritized vaccinations according to many vaccine protocols owing to a higher risk of COVID-19 infections and complications. Participants with comorbidities are assumed to have depleted responses to immunogens; thus, they are more susceptible to experiencing reduced side effects following any Vaccination [42]. In this study, participants with comorbid diseases were at higher risk of developing post-vaccination side effects, including increased blood pressure, heart rate, shortness of breath, voice hoarseness, dizziness, nausea, diarrhea, abdominal pain, and myalgia (muscle pain). These findings support the results of studies from the Arab region by Ma’mon M. Hatmal [31] and Saudi Arabia by Alghamdi et al. [43], which showed that chronic diseases are associated with developing post-vaccination side effects. However, further studies are needed to explain the causes of increased side effects following different COVID-19 vaccinations in comorbid people.

Regarding pre-vaccination COVID-19 infection, there was a significant association between previous COVID-19 infection and post-vaccination adverse effects. Participants who had COVID-19 infection before vaccination experienced higher post-vaccination side effects. This result was consistent with a multinational study among Arab populations and an Italian study [15, 32]. In this scenario, the natural vaccine infection of COVID-19 simulates the first vaccination dose, and side effects are more common after the second dose of the vaccine in patients who have never been infected. Thus, the first dose of the vaccine may serve as a booster dose in previously infected COVID-19 patients, causing an ADR to occur more frequently.

The CDC has developed training modules for healthcare workers (HCW) delivering the Pfizer-BioNTech (COMIRNATY^®^) COVID-19 vaccine to ensure good HCW practice, professional vaccine administration, and storage, as well as to provide HCWs with scientific data regarding vaccine safety and efficacy [44]. Adherence to vaccine protocols in administration, counseling, and patient assessments and providing patients with vaccine information sheets are essential to prevent complications and allergic reactions and ensure patient safety. Unfortunately, many HCWs did not implement the COVID-19 vaccination guidance designed by vaccine manufacturers, increasing the risk of improper vaccination techniques of intramuscular percutaneous injection into the patient’s arm [45]. However, Reports of SIRVA with the mRNA COVID-19 vaccines are rare, and it is hard to specify how common SIRVA is with the COVID-19 vaccine [46].

Nonetheless, this finding suggests that the healthcare field should be more attentive to vaccine-related training and education for HCWs to guarantee vaccine administration safety and good clinical practice. Furthermore, prevent complication that leads to missed days of work or school. In this study, 23% of participants reported work or school absence for a couple of days due to Pfizer-BioNTech vaccine side effects, which could influence the economy in the long term. A similar finding was reported in a US survey “AstraZeneca and Pfizer COVID vaccine shots lead to missed work days.” [47].

### Strengths

With increasing studies on the side effects of COVID-19 vaccines worldwide, our study is the first to evaluate the side effects of the Pfizer-BioNTech vaccine among Palestinians with a large sample size (N = 1137) and high educational level**.** Furthermore, our study’s main strength was the lack of opportunity for non-response bias because most questionnaires were filled based on face-face interviews. In addition, open-ended responses were primarily used in the survey, while close-ended answers (Yes/No) were rarely used, expanding the participants’ information.

### Limitations

As the study was conducted at a university, a higher percentage of responses were received from young people aged 18–23 year old (students) compared to older people aged > 30 year old (university staff), the age groups were unevenly distributed, causing the participant proportions in different groups to be biased. Second, although only a tiny percentage of questionnaire responses were collected online via Google Forms, differences resulted from exposure, interpretation, or misclassification of side effects. Third, this study was a self-reported study based on participant perception of adverse events, which was not clinically evaluated or confirmed, and could be related to other factors besides the vaccine; therefore, this study was unable to make a causality assessment of serious events as recommended by the WHO. Furthermore, uncovering severe side effects and establishing a direct causal relationship will require further research and studies. Therefore, further studies are recommended to cover the entire country, including the occupied Palestinian territories, to confirm the initial results of this study.

## Conclusion

COVID vaccines have been developed at an accelerated pace, and vaccine safety is a top priority; in this study, the most common post- BNT162b2 Vaccination reported self-limiting side effects similar to those reported by Pfizer/BioNTech Company. However, higher rates of allergic reactions were reported in this sample. In addition, rare side effects, such as thrombocytopenia and myocarditis, were reported by 8 participants. Therefore, standard monitoring through a national adverse event reporting system is necessary for safety assurance; continuous monitoring and long-term studies are required to ensure long-term vaccine safety.

## Supplementary Information

**Additional file 1.** Study Questionnaire.

## Data Availability

The datasets used and/or analyzed during the current study available from the corresponding author on reasonable request.
