# Supplementary material for: Side effects of Pfizer/BioNTech (BNT162b2) COVID-19 vaccine reported by the Birzeit University community
Source: BMC Infect Dis. 2023 Jan 5;23:5. doi: 10.1186/s12879-022-07974-3 (PMC9814351; doi:10.1186/s12879-022-07974-3)
Supplement: Supplementary file 1 — Additional file 1. Study Questionnaire. [file 12879_2022_7974_MOESM1_ESM.docx]

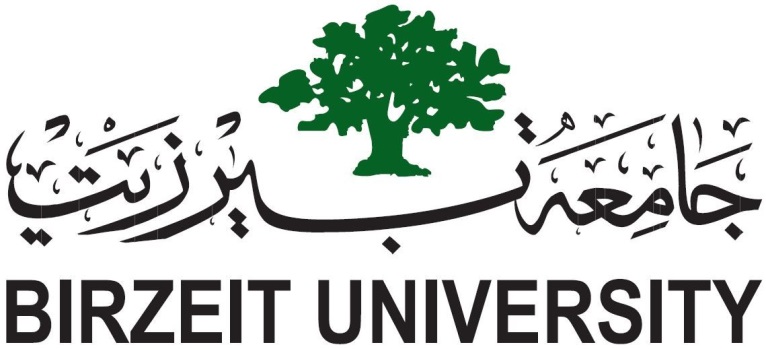


**Side effects of COVID-19 Vaccines reported by staff, students, and employees at Birzeit University.**

**Section 1: Participant consent**

Thank you for using your valuable time to complete this survey on the Side effects of COVID-19 Vaccines reported by staff, students, and employees at Birzeit University. Your participation in this questionnaire is voluntary. Your choice of the “Yes” option constitutes your consent to participate in this research; by participating in this study, you will help the researchers identify the side effects associated with COVID-19 vaccines. The information you are about to provide is for research purposes only. The survey will take approximately 5 -10 minutes to complete. We will not ask for your personal information, your responses will be kept confidential, and your identity will never be revealed. Your help is greatly appreciated.

If you have any questions or comments about filling this questionnaire, please contact Dr. Abdallah Abukhalil at [adkhalil@birzeit.edu](mailto:adkhalil@birzeit.edu).

| - No | - Yes | Do you agree to participate in this study? |
| --- | --- | --- |

**Section 2: Participant Information**

| - Female | - Male | Gender | |
| --- | --- | --- | --- |
|  | | | Age |
|  | | | Height |
|  | | | Weight |
| - Student | - University employee | Are you? | |

- **Are you suffering from any chronic disease? (You can choose one or more)**

| No |  |
| --- | --- |
| Diabetes Mellitus |  |
| Hypertension |  |
| Cardiovascular Disease |  |
| Lung disease (Asthma or COPD) |  |
| Rheumatism |  |
| Osteoporosis |  |
| Autoimmune disease |  |
| Cancer |  |
| Depression |  |
| Thyroid disease |  |

| - No | | - Yes | | | - **Do you have an allergy to any foods or medications?** |
| --- | --- | --- | --- | --- | --- |
| - No | | - Yes | | | - **Have you ever had an anaphylaxis reaction before covid-19 vaccination?** |
| - BOTH ( cigarette and shisha ) | - yes , hookah (shisha ) | | - Yes , cigarette | - No | - **Are you a smoker?** |

**Section3: Covid-19 Infection before vaccination**

- **Have you been infected with covid-19 before vaccination?**

| - No | - Yes |
| --- | --- |

- **If you have been infected with covid-19 before vaccination, how long did your symptoms last?**

| - More than seven days | - Less than 7 days |
| --- | --- |

- **If you have been infected with covid-19 before vaccination, were you hospitalized for COVID-19 treatment?**

| - No | - Yes |
| --- | --- |

- **If you were hospitalized for covid-19 infection before vaccination, if yes how long were you hospitalized?**

| - More than 14 days | - 7-14 days | - Less than 7 days |
| --- | --- | --- |

**Section 4: Covid-19 vaccination**

- **Which type of covid-19 vaccines have you received?**

| Pfizer / BioNTech |  |
| --- | --- |
| Moderna |  |
| Sputnik V |  |
| Sputnik light |  |
| Sinopharm |  |
| Astrazeneca/ Oxford |  |

- **How many doses have you received so far?**

| - Three doses | - Two doses | - Single dose |
| --- | --- | --- |

- **Have you been counseled by your healthcare provider about any possible side effects after receiving the vaccine?**

| - No | - Yes |
| --- | --- |

| - No | - Yes |
| --- | --- |
| - Skin rash and itching - Angioedema - Respiratory distress (such as shortness of breath or wheezing) - Coughing - Significant swelling of the tongue or lips | |

- **Did you have an allergic reaction following the covid-19 vaccination?**
- **What type of allergic reaction you had experienced?**

**Section 5: Adverse effects following vaccination**

- **Please fill the boxes with post-vaccination symptoms you have experienced?**

| Three doses | Two doses | First dose |  |
| --- | --- | --- | --- |
|  |  |  | NO symptoms |
|  |  |  | Fever |
|  |  |  | Chills |
|  |  |  | Headache |
|  |  |  | Hypertension |
|  |  |  | Increased Heart Rate |
|  |  |  | Shortness of breath |
|  |  |  | Persistent cough |
|  |  |  | Chest pain |
|  |  |  | Voice hoarseness |
|  |  |  | Dizziness |
|  |  |  | Ringing in the ears(Tinnitus) |
|  |  |  | Nausea |
|  |  |  | Vomiting |
|  |  |  | Diarrhea |
|  |  |  | Abdominal pain |
|  |  |  | Tiredness and fatigue |
|  |  |  | Pain or swelling at the injection site |
|  |  |  | Muscle pain ( myalgia ) |
|  |  |  | Joint pain |
|  |  |  | Swollen ankles and feet |
|  |  |  | Swollen armpit glands |
|  |  |  | Oversleepness and decrease sleep quality |
|  |  |  | Menstrual cycle changes ( if you are female ) |

If you experienced any other symptoms not listed above, please write them down? (Optional)_______________.

| - No | - Yes | Did you suffer from Shoulder injury related to vaccine administration?  Shoulder pain that began within 48 hours of vaccination, along with restricted movement in your shoulder |
| --- | --- | --- |

- **How soon did the symptoms appear after injection with a COVID-19 vaccine?**

| Third dose | Second dose | First dose |  |
| --- | --- | --- | --- |
|  |  |  | within first 12 hours |
|  |  |  | 12-24 h |
|  |  |  | 24-48 h |
|  |  |  | After 2 days |
|  |  |  | After one week or later |

- **How long did the symptoms last?**

| Third dose | Second dose | First dose |  |
| --- | --- | --- | --- |
|  |  |  | less than one day |
|  |  |  | 1-3 days |
|  |  |  | 4-7 days |
|  |  |  | more than week |

- **How many days of school or work did you miss because of vaccine side effects?**

| - More than two days | - Two days | - One day | - No school/ working days were missed |
| --- | --- | --- | --- |

- **How did you act to relieve symptoms that appeared after receiving the vaccine?**

| - Admitted to a hospital | - Went to the clinic without the need for hospitalization | - Rest at home and taking painkillers | - Only rest at home |
| --- | --- | --- | --- |

**Side effects that necessitate a doctor's visit**

Kindly fill the symptoms that led to a doctor's visit or hospitalization: ___________________________________

| - No | - Yes | - Have you been diagnosed with myocarditis and/or pericarditis after Covid-19 Vaccination? |
| --- | --- | --- |
| - No | - Yes | - Have you been diagnosed with any types of thrombosis (blood clots) After Vaccination? |
| - No | - Yes | - Have you been diagnosed with low platelet count (thrombocytopenia) after vaccination? |

**Section 6: Covid-19 Infection Post vaccination**

| Yes , More than 2 months after the vaccine | Yes , between 1 -2 months after the vaccine | Yes , less than one month after the vaccine | No | - Have you got infected with COVID-19 after vaccination? |
| --- | --- | --- | --- | --- |

| - More than seven days | - Less than 7 days | | - If you have been infected with covid-19 after vaccination, how long did your symptoms last? |
| --- | --- | --- | --- |
| - No | - Yes | | - If you have been infected with covid-19 after vaccination, were you hospitalized for COVID-19 treatment? |
| - More than 14 days | - 7-14 days | - Less than 7 days | - If you were hospitalized for covid-19 treatment, if yes how long were you hospitalized? |
